# Chronic Dexamethasone Disturbs the Circadian Rhythm of Melatonin and Clock Genes in Goats

**DOI:** 10.3390/ani15010115

**Published:** 2025-01-06

**Authors:** Liuping Cai, Qu Chen, Canfeng Hua, Liqiong Niu, Qijun Kong, Lei Wu, Yingdong Ni

**Affiliations:** 1 Jiangsu Key Laboratory of Sericultural Biology and Biotechnology, School of Biotechnology, Jiangsu University of Science and Technology, Zhenjiang 212100, China; m15295517060@163.com (L.C.); 17826079131@163.com (Q.K.); 2 Key Laboratory of Animal Physiology & Biochemistry, College of Veterinary Medicine, Nanjing Agricultural University, Nanjing 210095, China; 2016107018@njau.edu.cn (Q.C.); huacanfeng@hotmail.com (C.H.); 2016107017@njau.edu.cn (L.N.); 15251818217@163.com (L.W.)

**Keywords:** dexamethasone, melatonin, gut, biological rhythm, ruminant

## Abstract

Dex is a drug commonly used as an immunosuppressive and anti-inflammatory agent in humans and animals. GCs have a profound impact on melatonin expression and biological rhythm. However, the effect of chronic exposure to Dex on melatonin secretion and biological clock gene expression in ruminants is still unclear. Ten goats were randomly divided into two groups: the control group was injected with saline, and the Dex-treated group was intramuscularly injected daily for 21 d with 0.2 mg/kg Dex. The rhythm of melatonin secretion in the plasma was disturbed in the Dex group, and the plasma and colon levels of melatonin were lower in the Dex group compared to the control group (*p* < 0.05). Dex leads to a significant decrease in the expression of Arylalkylamine N-acetyltransferase (AANAT), a key melatonin synthase, in the pineal gland and colon. Detecting intestinal leakage-related indices showed that diamine oxidase (DAO) and lipopolysaccharide (LPS) content increased significantly in the Dex group (*p* < 0.05). We also detected genes associated with biological rhythms in the plasma. In the control group, the five tested genes showed circadian rhythms, but the circadian rhythms of Clock, Cry1, Cry2, and Per2 were abolished or blunted by the Dex (*p* < 0.05). Protein levels of CLOCK and BMAL1 in the colon changed significantly (*p* < 0.05). In conclusion, the above experimental results show that chronic exposure to Dex leads to the disorder of the circadian rhythms of melatonin secretion and clock genes.

## Simple Summary

Dexamethasone (Dex), an artificially synthesized glucocorticoid (GC), is commonly used as an immunosuppressive agent and anti-inflammatory agent. Long-term Dex treatment can cause negative effects. Melatonin not only regulates biological rhythms but also plays an important role in maintaining intestinal health in the gut. In this study, the long-term intramuscular injection of low-dose Dex was used to observe the changes in the circadian rhythm of melatonin and clock genes and the intestinal leakage-related indexes in goats.

## 1. Introduction

Melatonin, produced and released by the pineal gland of vertebrates, is a hormone secreted predominantly during the nighttime portion of the daily light–dark cycle. It plays a pivotal role in synchronizing the organism’s internal biological rhythm with the extrinsic light–dark cycles. By being secreted during darkness, melatonin conveys signals to the primary circadian pacemaker, the suprachiasmatic nucleus (SCN), located in the hypothalamus, which then regulates sleep–wake cycles and other daily bodily functions. Future studies should concentrate on uncovering the intricate molecular mechanisms that govern melatonin’s functions, especially in complex physiological and disease states [1]. Melatonin serves multiple functions: the maintenance of circadian rhythms and the prevention of obesity, neurodegenerative diseases, and viral infections, but also the modulation of the gut microbiome and the enhancement of gut health [2,3]. Recent studies have found that the intestine, a vital melatonin repository, can synthesize and secrete a large amount of melatonin [4]. In the intestine, melatonin is essential in maintaining intestinal microbial homeostasis, intestinal mucosal immunity, and epithelial cell metabolism [2]. The synthesis of melatonin can be modulated by GCs, as evidenced by increased levels of GCs leading to disruptions in the biological clock of mice [5]. Moreover, studies in humans and mice showed that sleep deprivation leads to decreased intestinal melatonin, increased intestinal GCs, damaged intestinal barrier, and microbiome dysbiosis [6,7,8].

A variety of endogenous compounds, including GCs, have been proposed to impact melatonin production across different vertebrate species [9,10,11]. For instance, high GC levels, often triggered by stress, can suppress melatonin production, potentially disrupting the normal circadian rhythm and leading to sleep disturbances [5,12]. However, the effect of chronic exposure to Dex on melatonin secretion in goats has not been reported.

GCs directly regulate the circadian clock. All animals have a physiological mechanism called the “biological clock”, a 24 h cycle rhythm from day to night. The metabolic activities of life have their biological rhythms, such as hormone secretion, nutrient absorption and metabolism, gene transcription, and so on [13]. At the cellular level, mammalian circadian clocks function through two interconnected transcriptional feedback loops, which seamlessly regulate the expression of their components by either enhancing or suppressing their activity. This feedback loop consists of periods (Per1, Per2, and Per3), cryptochromes (cry1 and cry2), and circadian locomotor output cycles kaput (Clock)/brain and muscle ARNT-like (BMAL1). Intestinal typical physiological function is crucially maintained by the biological clock. The essential clock gene BMAL1 can modulate the regeneration of intestinal epithelial cells by influencing cytokines, cell cycle progression, and cellular proliferation [14]. The deletion of the Per2 gene in mice resulted in a downregulation of intestinal tight junction proteins and consequently enhanced intestinal permeability [15]. It is worth studying whether chronic exposure to Dex influences the biological clock in goats, which may impact intestinal function. Hence, this experiment aims to find out the effect of chronic exposure to Dex on melatonin secretion’s circadian rhythm, clock gene expression, and intestinal barrier function in goats.

## 2. Materials and Methods

The experimental protocol, bearing project number 31972638, was approved by the Animal Ethics Committee of Nanjing Agricultural University. The sampling procedures were conducted in accordance with the “Guidelines on Ethical Treatment of Experimental Animals” (No. 398, 2006) issued by the Ministry of Science and Technology of China.

### 2.1. Animals and Experimental Design

In summary, ten healthy male goats, each weighing approximately 25 kg (with a variation of ±1 kg), were fitted with ruminal cannulas at Nanjing Agriculture University. The goats were arbitrarily divided into 2 groups. Every goat was housed separately and given unrestricted access to water. The goats were purchased from Jiangsu Taizhou Xilaiyuan Co., Ltd. The goats were fed with forage-based diets (Alfalfa hay-*Medicago sativa* L.) and a concentrated mixture (the diet comprised 12% oats, 15% faba beans, 25% barley, 10% peas, 20% sugar beet pulp, 5% molasses, and 3% mineral and vitamin supplements, amounting to a total of 250 g per animal) at 8:00 a.m. and 6:00 p.m. from October to December. The goats in the control group received intramuscular saline injections, while the Dex group received intramuscular injections of Dex (0.2 mg/kg) before 8:00 a.m. Each goat was clinically healthy, exhibiting no signs of disease or parasites. Their overall health was thoroughly assessed using a range of indicators, including body temperature, cardiac rate, respiratory rate, appetite levels, fecal consistency, and hematologic indices. The experiments were conducted under natural light conditions. One day prior to the experiment, polyethylene catheters were sterilely put into the external jugular vein of each goat. Blood samples were taken every four hours for a continuous 24 h period, commencing at 04:00 on day 20 and concluding at 04:00 on day 21. The collected samples were subsequently transferred into PAX gene Blood RNA Tubes provided by Qiagen (Shanghai, China) and kept at −80 °C for future analysis.

On day 21, after fasting overnight, all the goats were euthanized through injections of xylazine (administered at a dosage of 0.5 mg/kg of body weight; brand name Xylosol, manufactured by Ogris Pharme in Wels, Austria) and pentobarbital (50 mg/kg of body weight; brand name Release, manufactured by WDT in Garbsen, Germany). All the tissue samples were collected and stored at −80 °C. The materials and methods are similar to the author’s previously published article [16].

### 2.2. Measurement of Plasma and Colon Melatonin and Plasma Cortisol Level

Using the melatonin ELISA kit (ENZ107 KIT150-0001, Enzo Lifesciences, Plano, TX, USA) following the manufacturer’s guidelines, the plasma melatonin levels were measured. The colon contents were blended with 500 µL alcohol, followed by centrifugation to separate the supernatant, which was then used for melatonin detection. The assay exhibited a sensitivity of 0.08 pg/mL, covering a range from 1 to 162 pg/mL, with an intra-assay coefficient of variation below 15%. Each sample was tested twice. The cortisol assay had a sensitivity of 1 ng/mL and demonstrated an intra-assay coefficient of variation of 7.0%. To maintain consistency and eliminate inter-assay variability, all the samples were tested within a single batch.

### 2.3. Assay of Arylalkylamine N-Acetyltransferase (AANAT)

Colonic epithelial tissue and pineal gland tissue were collected and weighed (9 g/sample), respectively. The samples were homogenized in the designed buffer provided in AANTA ELISA KIT (Shanghai GuYan Industrial Co., Ltd., Shanghai, China) and subjected to extract protein. The melatonin synthase enzyme content was measured according to the kit’s instructions.

### 2.4. Assay of Malondialdehyde and Glutathione Content

The blood samples were stored at −20 °C for subsequent Malondialdehyde and Glutathione analysis. The MDA and GSH levels were determined using ELISA kits provided by Beijing Solarbio Science and Technology Co., Ltd. (Beijing, China), adhering to the manufacturer’s guidelines.

### 2.5. RNA Isolation, cDNA Synthesis, and Real-Time PCR

Whole blood samples from the goats were used to directly extract Total RNA utilizing the PAX Gene Blood RNA kit (Qiagen) as per the manufacturer’s guidelines. The extracted RNA was then resuspended in 80 milliliters of Elution Buffer. Following this, mRNA was reverse-transcribed as per the manufacturer’s directions. For real-time PCR, performed on the Mx3000P instrument (Stratagene, Cedar Creek, TX, USA), 2 microliters of diluted cDNA (1:40, *v*/*v*) was used, with GAPDH acting as the reference gene. The results from the real-time PCR were analyzed employing the 2^−ΔΔCt^ method, while all the primers listed in Table 1 were synthesized by Tsingke Company, located in Nanjing, China. The materials and methods are similar to the author’s previously published article [16].

### 2.6. Assay of DAO and LPS

The concentration of DAO was detected by Micro DAO (Beijing Solarbio Science and Technology Co., Ltd.). The operation process was carried out in strict accordance with the instructions. The concentration of LPS was detected by a Lipopolysaccharide (LPS) ELISA Kit (Cloud-Clone Corp., formerly Uscn Life Science Inc., Wuhan, China). The minimum detectable dose of sensitivity is less than or equal to 0.19 ng/mL. The operation process was carried out following the instructions strictly.

### 2.7. Western Blotting Analysis

Each sample’s protein, amounting to thirty micrograms, was separated using 10% SDS-PAGE gels and then transferred onto nitrocellulose membranes (Bio Trace; Pall Corp., New York, NY, USA). Following the aforementioned step, the membranes were incubated in a blocking buffer for two hours at room temperature to prevent non-specific binding. After that, they were kept at 4 °C overnight for incubation with primary antibodies: rabbit-anti-GR (1:1000; DF4994; Protech, Cambridge, UK), CLOCK (1:1000; ab65033; Abcam, Cambridge, UK), BMAL1 (1:1000; ab228594; Abcam, Cambridge, UK), and anti-Tubulin-α (1:10,000; BS1699; Bioworld, Dublin, OH, USA). After washing with 1x PBS, the membranes were then exposed to goat anti-rabbit HRP-conjugated IgG (diluted 1:10,000; Bioworld) for a duration of 2 h at room temperature. Utilizing an imaging system from Bio-Rad (Hercules, CA, USA), the blot was visualized, and the intensity of the bands was subsequently analyzed using the Quantity One 1-D software, also provided by Bio-Rad (Hercules, CA, USA). The materials and methods are similar to the author’s previously published article [16].

### 2.8. Statistical Analysis

The data are reported as the average ± standard error of the mean (SEM). Using the IBM SPSS Statistics 20 software (Armonk, New York, NY, USA), statistical analyses were conducted by *t*-test and one-way ANOVA.

The goal of these analyses was twofold: First, to determine the statistical significance of the differences across the six daily time points and confirm the existence of daily fluctuations (with a significance level of *p* ≤ 0.05), thereby laying the foundation for the subsequent cosinor analysis. Second, to evaluate the circadian rhythmicity, the mRNA levels of clock-related genes were analyzed separately using MATLAB 7.0 (from MathWorks Inc., Natick, MA, USA) by applying unimodal cosinor regression [y = A + (B × cos (2π(x − C)/24))], where y denotes the mRNA level at a given time x. A, B, and C represent the mesor, amplitude, and acrophase, respectively. The statistical significance of regression analysis results was determined by taking into account elements like sample size, R2 values, and the number of predictors (mesor, amplitude, and acrophase). A *p*-value threshold of ≤0.05 was set for this determination. To compare the differences in mesor, amplitude, and acrophase between the Con and Dex groups, a one-way ANOVA was carried out, and subsequently, Fisher’s LSD post hoc test was employed.

## 3. Results

### 3.1. Effect of Chronic Dex Exposure on the Concentrations of Melatonin and Cortisol in Goats

**Figure 1:**
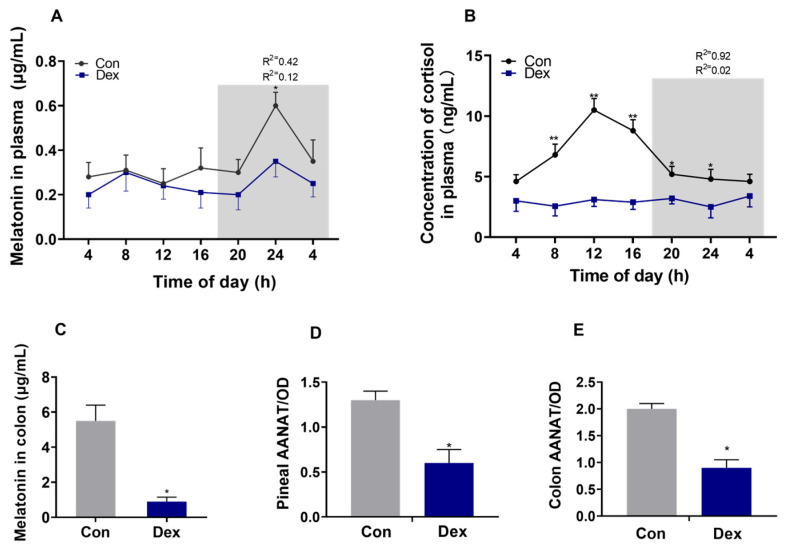
Effect of chronic Dex exposure on the melatonin and cortisol content. (**A**) The concentration of melatonin in the plasma; (**B**) the concentration of cortisol in the plasma. (**C**) The melatonin content in the colon; (**D**) the level of AANTA in the pineal gland; (**E**) the level of AANTA in the colon. n = 5 goats per time point. The degree of fitting is represented by the R^2^ values. Data are mean ± SEM; * *p* < 0.05 and ** *p* < 0.01 compared with Con.

The levels of melatonin (Figure 1A) and cortisol (Figure 1B) in the control group showed changes in diurnal patterns (*p* < 0.05, one-way ANOVA), which was disturbed in the Dex group. The results of the analysis of colon contents indicated that the Dex treatment resulted in a decrease in melatonin (*p* < 0.05) in the colon compared with the Con treatment on day 21 (Figure 1C). Meanwhile, a significant reduction (*p* < 0.05) in AANAT, a key enzyme in melatonin production, was observed in the pineal gland and colon tissue samples (Figure 1D,E). The mesors of cortisol and melatonin content were markedly downregulated (*p* < 0.05) by the Dex treatment, and the amplitude of melatonin content was decreased by Dex (*p* < 0.05) (Table 2).

### 3.2. Effect of Chronic Dex Exposure on the Circadian Rhythm of Clock Genes in Plasma

**Figure 2:**
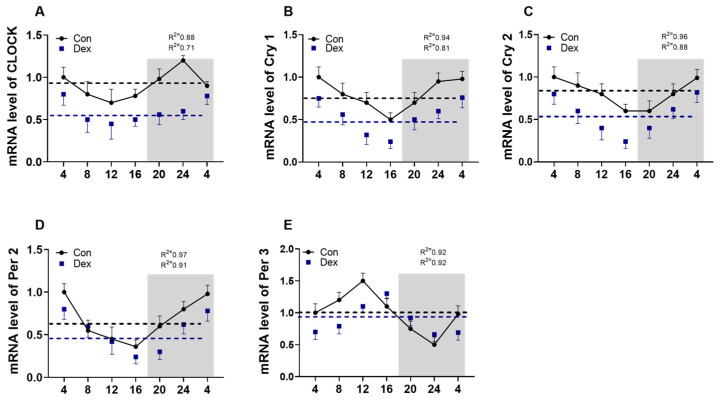
The circadian rhythm parameters of clock genes in the plasma. (**A**) Clock genes; (**B**) Cry1 gene; (**C**) Cry2 gene; (**D**) Per2 gene; (**E**) Per3 gene. In the graphs, the data markers denote the mRNA expression levels of clock genes. The curves illustrate the 24 h period identified by cosinor analysis. There are 5 goats for each time point (n = 5). The R^2^ values signify the degree of fitting. The values are in the form of mean ± SEM.

In the Con group, circadian rhythms were exhibited in all five of the tested genes. However, the circadian rhythms of Clock, Cry1, Cry2, and Per2 were either eliminated or weakened upon the administration of Dex (*p* < 0.05, one-way ANOVA). Dex significantly reduced the mesor of mRNA of Clock, Cry1, Cry2, and Per2 (Table 3). In Figure 2E, the peak of mRNA of Per3 during the day was delayed by four hours in the Dex group. In Table 3, the acrophase of mRNA of Cry2 was decreased by Dex, but the acrophase of mRNA of Per2 was increased by Dex (*p* < 0.05, one-way ANOVA).

### 3.3. Effect of Chronic Dex Exposure on the GSH and MDA in Plasma

**Figure 3:**
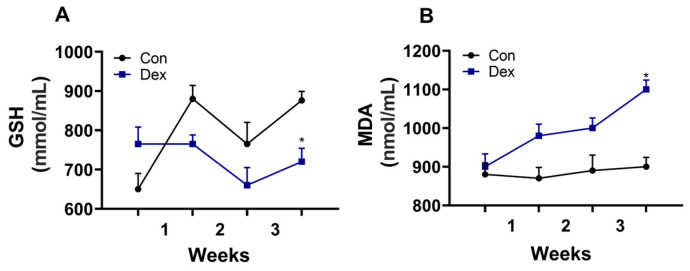
Effect of chronic Dex exposure on the concentration of GSH and MDA in the plasma. (**A**) GSH content; (**B**) MDA content. n = 5 goats, and the values are mean ± SEM; * *p* < 0.05 compared with Con.

GSH levels began to decrease after one week of Dex treatment and were significantly different from the Con at day 21 (*p* < 0.05, *t*-test) (Figure 3A). MDA content showed an increasing trend at the beginning of the experiment, and a remarkable disparity emerged between the Dex treatment group and the control group at day 21 (*p* < 0.05, *t*-test) (Figure 3B).

### 3.4. Effect of Chronic Dex Exposure on Intestinal Barrier Function Indexes (DAO and LPS)

**Figure 4:**
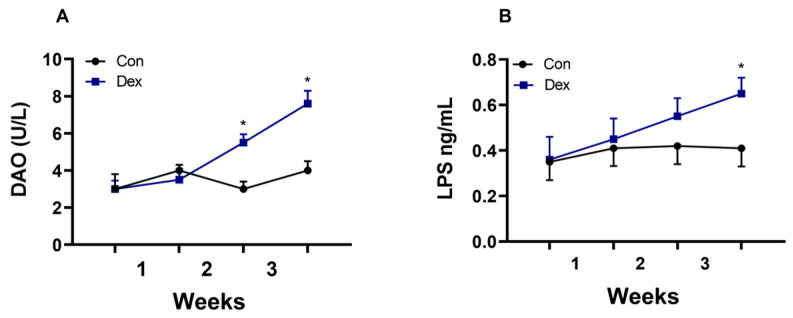
Effect of chronic Dex exposure on the concentration of DAO and LPS in the plasma. (**A**) DAO content; (**B**) LPS content. n = 5 goats, and the values are mean ± SEM, * *p* < 0.05 compared with Con.

The results indicated that the level of DAO in the Dex group exceeded that in the Con group (*p* < 0.05, *t*-test) in the last week of the experiment (Figure 4A). Also, on day 21, the results indicated that the LPS level in the Dex group was greater than that in the Con group. (*p* < 0.05, *t*-test) (Figure 4B).

### 3.5. Effect of Chronic Exposure to Dex on CLOCK\BMAL1 and GR Protein Expression

**Figure 5:**
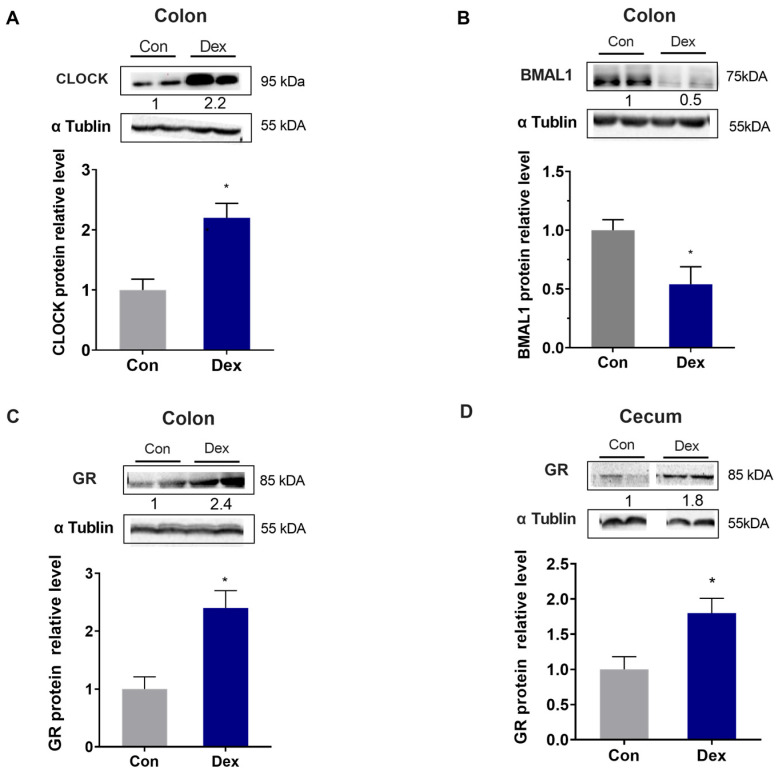
Effect of chronic Dex exposure on the protein expression of CLOCK, BMAL1, and GR. (**A**) CLOCK protein expression in the colon; (**B**) BMAL1 protein expression in the colon; (**C**) GR protein expression in the colon; (**D**) GR protein expression in the cecum. n = 5 goats, and the values are mean ± SEM; compared with the Con, * *p* < 0.05.

Through further analysis of the protein expression in the colon and cecum, we found that the chronic Dex treatment markedly enhanced the expression of CLOCK protein and reduced the expression of BMAL1 protein. (*p* < 0.05, *t*-test) (Figure 5A,B). Furthermore, the expression of GR in the colon and cecum of the treatment group was substantially elevated in comparison with that of the control group. (*p* < 0.05, *t*-test) (Figure 5C,D). The original western blot images are in Supplementary Figure S1.

## 4. Discussion

In this study, prolonged exposure to the Dex treatment resulted in the disruption of the goats’ melatonin circadian pattern in the plasma, suggesting a disturbance in their internal biological clocks. Additionally, it was observed that the Dex treatment resulted in a reduction in cortisol levels among the mammals. Previous studies have reported that GCs exert a feedback inhibitory effect on their secretion by hindering the corticotrophin-releasing hormone’s action on the pituitary gland [17,18]. Notably, our study observed a notable suppression of natural cortisol production due to the Dex treatment. The findings suggest the successful establishment of an experimental model for chronic GC administration in goats.

In order to investigate the cause of the decrease in melatonin, we detected the critical enzyme of melatonin synthesis, and the findings indicated that the amount of AANAT synthesis was reduced in the pineal gland. Therefore, long-term exposure to Dex is associated with a decrease in AANAT synthesis. In mice, chronic exposure to Dex is associated with a decrease in melatonin and critical enzymes of melatonin synthesis [5], which is in line with our results. Previous studies showed that MDA and GSH are affected by melatonin [13], and in our results, a decrease in plasma melatonin also leads to a decrease in GSH and an increase in MDA in the plasma.

In our experiment, we also found that the long-term injection of Dex led to a decrease in the secretion of melatonin in the intestine. Melatonin in the intestine is crucial for maintaining normal intestinal function and immunity. The melatonin concentration may be associated with an imbalance in intestinal microbiota, increased inflammatory factors, and even a change in intestinal barrier permeability [8,19]. The data showed that AANAT synthesis decreased in colon tissue, which caused a decrease in melatonin. Previous studies have also shown that insufficient melatonin secretion in the gut leads to intestinal mucosal damage and microbial disturbance [2]. In our published data, it has been shown that long-term Dex treatment leads to an increase in intestinal inflammatory factors and the abnormal expression of genes and proteins of tight junction proteins [16]. Our experimental results showed that DAO and LPS in the plasma increased, indicating that intestinal barrier function might be impaired [20].

Studies have shown that the disruption of biological rhythms can cause damage to the intestinal barrier and is associated with many intestinal diseases [21]. Previous research has indicated that disruptions in the internal clock mechanisms can result in the development of metabolic diseases and cerebrovascular diseases [22,23]. In our work, the impact of chronic Dex exposure on the expression of circadian clock-related genes in ruminants was investigated. In recent years, there has been an increase in research examining the patterns of clock genes in peripheral blood, which, along with a handful of other studies, have shown that these genes exhibit oscillatory behavior not only within the central nervous system but also extending to the peripheral organs and blood [24,25]. The mRNA expression of these genes in whole blood has been noted to vary over time in mammals. Specifically, the gene expression of Per2, Bmal1, Cry2, and Clock reaches its peak during the early night in cows [26,27]. The transcriptional/translational feedback loop involving genes like clock, cry1, cry2, per1, and per2 in the regulation of the biological clock is very important. Our study revealed a consistent daily pattern in all the examined clock gene expression in the blood. Notably, each gene showed a prominent disparity in the mesor level and rhythm amplitude compared to the others. Except for Per3, all the rhythmic genes demonstrated a nocturnal acrophase. It is possible that chronic Dex exposure is associated with the daily rhythms of all the key clock genes in the blood. In the Dex group, a four-hour delay occurred in the daytime peak of Per 3 compared to the control group. Blood consists of various cell types that are not synchronized, explaining the lack of reciprocal expression of positive and negative clock elements in peripheral circadian oscillators [28,29,30].

The experimental subjects in this article are male goats, and there is still a lack of research on the impact of gender in this research. Studies have shown that Dex affects the expression of isoforms of the GR in different genders [31]. In addition, there are also differences in the expression and phosphorylation levels of the GR among different genders [32]. Therefore, in this experiment, the impact of chronic exposure to Dex on female goats may be different from that on male goats, which is worthy of further research.

## 5. Conclusions

It is plausible that Dex directly modulates the expression of clock genes through GR-mediated transcriptional regulation, as suggested in previous studies [33]. Additionally, melatonin serves as a vital regulator in controlling circadian rhythms [34]. Our study observed that the disruption of the plasma melatonin circadian rhythm corresponded to the attenuation of the circadian pattern of clock genes. Therefore, we hypothesize that chronic Dex exposure may indirectly affect the rhythmic expression of circadian clock genes by altering melatonin secretion.

## Figures and Tables

**Table 1:** Primer sequences for RT-PCR.

Target Genes	Primer Sequences 5′–3′	Source
PER1	F: CTCTTCCTGCACTGCCTCTT	XM_018042917.1
	R: TGATGATGTCTTTCTTGGCAC	
PER2	F: AGCGTCAGGATGACCTACCA	XM_018064614.1
	R: GTCCTCTGGCCTCACAGTTT	
CLOCK	F: TTCGACAGGACTGGAAACCT	XR_001917237.1
	R: CTTCCATCTGTCATGATTGCTC	
CRY1	F: TCCGCTGCGTCTACATCCT	NM 001129735.1
	R: CAAAAATCGCCACCTGTTGA	
CRY2	F: CAGGAAGGTGAAGCGGAACA	NM 001129736
	R:TAAAAGAACTCTCGCCACAGAAGTT	
DAPDH	F: GGGTCATCATCTCTGCACCT	HM043737.1
	R: GGTCATAAGTCCCTCCACGA	

**Table 2:** Circadian rhythm parameters of melatonin determined by cosinor analyses.

Index	Group	Melatonin	Cortisol
Mesor	Con	0.34 ± 0.01	6.77 ± 0.82
	Dex	0.25 ± 0.02 *	2.95 ± 0.62 **
Amplitude	Con	0.11 ± 0.02	ND
	Dex	0.03 ± 0.03 **	0.02 ± 0.01
Acrophase, h	Con	ND	0.99 ± 0.32
	Dex	3.5 ± 0.17	ND

The values are means ± SEM. * *p* < 0.05 and ** *p* < 0.01 compared with the Con group. ND stands for not determined since there was no circadian rhythm.

**Table 3:** Circadian rhythm parameters of clock genes determined by cosinor analyses.

Index	Group	Clock	Cry1	Cry2	Per2	Per3
Mesor	Con	0.89 ± 0.04	0.77 ± 0.03	0.78 ± 0.27	0.61 ± 0.02	1.09 ± 0.76
	Dex	0.57 ± 0.02 *	0.49 ± 0.04 *	0.52 ± 0.18 *	0.40 ± 0.03 *	0.91 ± 0.52
Amplitude	Con	0.21 ± 0.02	0.22 ± 0.04	0.21 ± 0.12	0.30 ± 0.06	ND
	Dex	0.15 ± 0.03	0.24 ± 0.03	0.27 ± 0.09	0.27 ± 0.04	ND
Acrophase, h	Con	ND	3.26 ± 1.32	5.65 ± 0.68	2.52 ± 0.58	ND
	Dex	2.4 ± 1.31	2.99 ± 0.98	3.91 ± 0.51 *	4.40 ± 0.65 *	2.81 ± 1.23

The values are means ± SEM. * *p* < 0.05 compared with the Con group. ND stands for not determined since there was no circadian rhythm.

## Data Availability

None of the data were deposited in an official repository. Data that support those study findings are available upon request.

## Supplementary Materials

The following supporting information can be downloaded at: https://www.mdpi.com/article/10.3390/ani15010115/s1, Figure S1: The original western blot images.
