# Supplementary figures and images for: Chronic Dexamethasone Disturbs the Circadian Rhythm of Melatonin and Clock Genes in Goats
Source: Animals (Basel). 2025 Jan 6;15(1):115. doi: 10.3390/ani15010115 (PMC11718956; doi:10.3390/ani15010115)

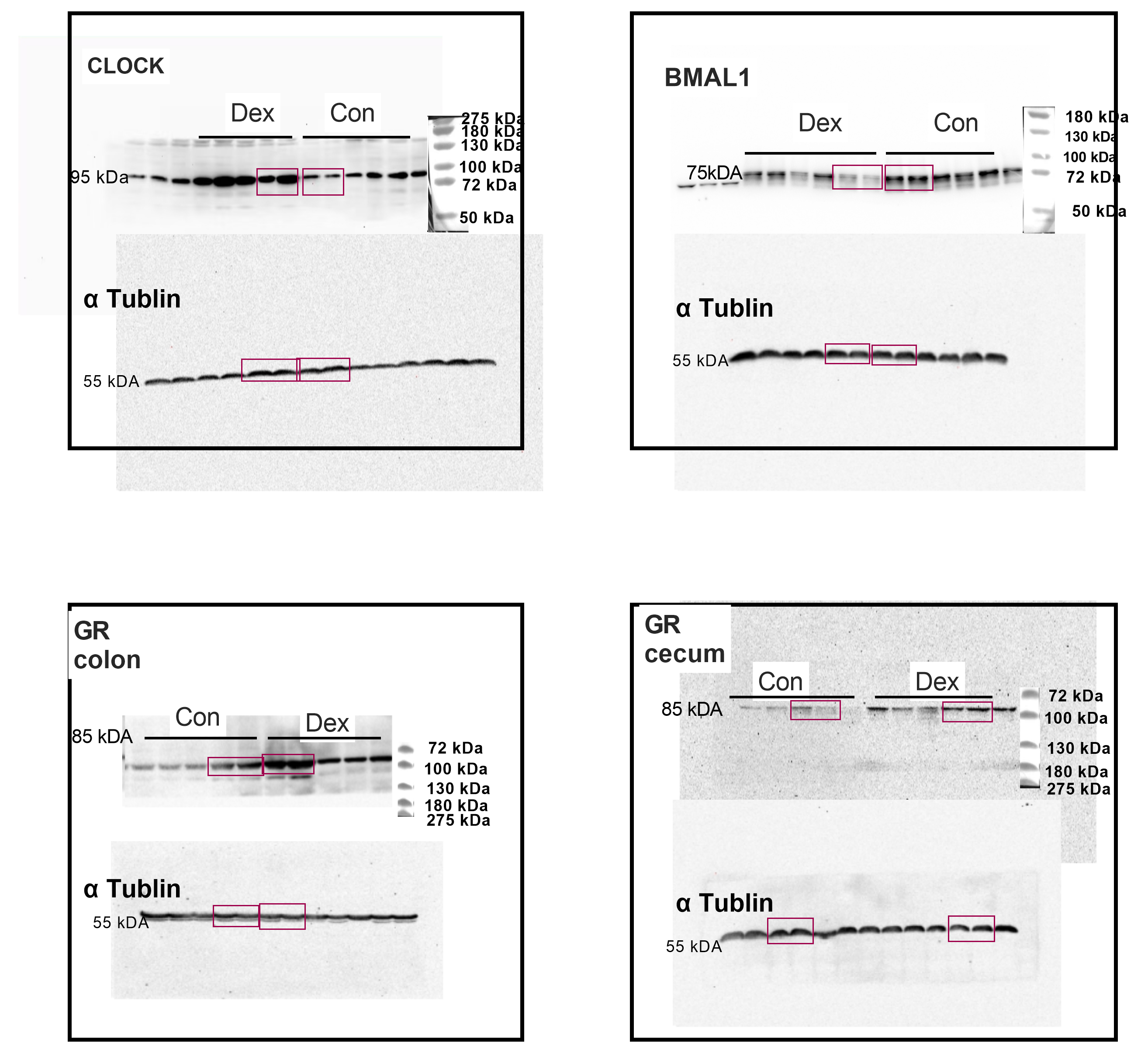

Supplement: Supplementary file 1 [file animals-15-00115-s001.zip › animals-3359778-supplementary.tif]
